# Estimation of Apigenin, an Anxiolytic Constituent, in *Turnera aphrodisiaca*

**DOI:** 10.4103/0250-474X.49143

**Published:** 2008

**Authors:** S. Kumar, R. Madaan, A. Sharma

**Affiliations:** S. D. College of Pharmacy, Barnala-148 101, India; 1 University Institute of Pharmaceutical Sciences, Panjab University, Chandigarh-160 014, India

**Keywords:** Anxiolytic, Aphrodisiac, Apigenin, HPTLC densitometry, *Turnera aphrodisiaca*

## Abstract

An HPTLC densitometric method has been developed to estimate apigenin in *Turnera aphrodisiaca* aerial and its segregated parts (leaves, stems, flowers and fruits) so that plant can be standardized on the basis of its bioactive marker. The apigenin content in methanol extract of *T. aphrodisiaca* aerial parts was found to be about fourteen times less than acid hydrolyzed methanol extract of the plant indicating the presence of most of apigenin in glycosidic form. Amongst different plant parts, flowers possessed maximum content of apigenin followed by leaves. The apigenin content was also determined in three marketed formulations of *T. aphrodisiaca* viz., NLK, DWSG and SBL. DWSG contained higher content of apigenin. Aerial parts of the plant were collected at bimonthly intervals over a period of one year in the months of January, March, May, July, September and November. The plant material collected in September showed maximum content of apigenin.

*Turnera aphrodisiaca* Ward (synonym *T. diffusa* Willd., family Turneraceae) is commonly known as Damiana. The leaves of *T. aphrodisiaca* have been used traditionally as a stimulant, aphrodisiac, tonic, diuretic, nerve tonic, laxative, and in kidney, menstrual and pregnancy disorders^1^. The British Herbal Pharmacopoeia^2^ lists specific indications for damiana as anxiety neurosis associated with impotency, and includes other indications such as depression, nervous dyspepsia, atonic constipation and coital inadequacy. Mother tincture (85% ethanol extract) of damiana is an important homoeopathic medicine for the treatment of sexual debility, and nervous prostration^3^. Phytochemical reports on *T. aphrodisiaca* indicate that the plant contains tetraphyllin B (cyanoglycoside)^4^, gonzalitosin I (flavonoid)^5^ and β-sitosterol (phytosterol)^5^.

A survey of literature on *T. aphrodisiaca* revealed sporadic pharmacological reports on the plant. Aguilara *et al.*^6^ have reported that decoction of *T. aphrodisiaca* leaves possesses significant hypoglycaemic activity in rabbits upon oral administration. Aqueous extract of the plant was reported to exhibit aphrodisiac activity in sexually sluggish male rats at a dose of 1 ml/kg^7^. Recently, we have reported that amongst various extracts viz., petroleum ether, chloroform, methanol and water of *T. aphrodisiaca* aerial parts, only methanol extract (25 mg/kg, p.o.) exhibited significant anti-anxiety activity on elevated plus maze apparatus^8^. An anxiolytic constituent apigenin has been isolated from methanol extract of *T. aphrodisiaca* aerial parts using bioactivity-guided fractionation^9^. Present investigation was undertaken with an objective to develop a HPTLC densitometric method to estimate apigenin in *T. aphrodisiaca* aerial and its segregated parts viz., leaves, stems, flowers and fruits so that plant can be standardized on the basis of its bioactive marker.

*T. aphrodisiaca* whole plant was procured from a cultivated source, Rati Ram Nursery, Village Khurrampur, district Saharanpur (UP) in the month of August 2002, and dried in shade. Identity of the plant was confirmed through Botanical Survey of India, Howrah. A voucher specimen (No. 314) of the plant has been deposited in the Herbarium-cum-Museum of the University Institute of Pharmaceutical Sciences, Panjab University, Chandigarh. The mother tinctures from three different manufacturers viz., National Laboratory, Kolkata, India (Batch No. 304, NLK), DHU-Arzneimittel, Germany (Batch No. 3821002, DWSG) and SBL Private Limited, Sahibabad, Ghaziabad, India (Batch No. MT321, SBL) were procured from the local market.

The HPTLC system consists of Linomat-IV applicator and Camag TLC Scanner – III with CATS 4 software. TLC plates were developed in TLC chamber (Merck) saturated (10 min) with toluene:ethyl acetate (1:4) as the mobile phase. The development length was 50 mm. The plates were dried and scanned at 336 nm. Stock solution of apigenin (1.25 mg/10 ml) was prepared in methanol. From this stock solution, four working standard solutions were obtained by appropriate dilution with methanol. The concentrations of working standard solutions were 1, 0.75, 0.5 or 0.25 mg/10 ml.

Dried powdered aerial parts of *T. aphrodisiaca* (1 g), packed in a filter paper sachet, were defatted by refluxing in 250 ml round bottom flask on boiling water bath with 3×50 ml quantity of petroleum ether (1 h each). The marc obtained was air dried and refluxed under similar conditions with 3×50 ml quantity of methanol. The methanol extracts were pooled, filtered and concentrated under reduced pressure. Dried methanol extract was reconstituted in methanol, in a volumetric flask, and its volume was made up to 10 ml.

Acid hydrolysis was performed following the procedure mentioned above, methanol extract of *T. aphrodisiaca* aerial parts was prepared. Dried methanol extract, thus obtained, was heated in a 50 ml round bottom flask with 6% aqueous hydrochloric acid (25 ml; E Merck) for 45 min on water bath in order to hydrolyze flavonoid *O*-glycosides^10^. Aglycones, precipitated on cooling the solution, were removed by filtration, and dissolved in methanol. The last traces of aglycones were removed from filtrate by extracting with 3×20 ml quantity of diethyl ether. Latter were combined, dried over anhydrous sodium sulphate, and evaporated under reduced pressure. The diethyl ether extract, thus obtained, was pooled with methanol solution of aglycones. Finally, volume was made up to 25 ml with methanol in a volumetric flask.

Segregated aerial parts viz., leaves, stems, flowers and fruits (1 g each) were extracted as per the procedure optimized, followed by acid hydrolysis described above. The final volume was made up to 25 ml (leaves), 10 ml (stems), 25 ml (flowers) and 10 ml (fruits) with methanol. Mother tinctures of *T. aphrodisiaca* (30 ml each) from NLK, SBL and DWSG were concentrated under reduced pressure, and dried extracts were acid hydrolyzed as per the procedure described above. The final volume was made up to 10 ml (NLK) and 50 ml (SBL and DWSG) with methanol. Aerial parts of *T. aphrodisiaca* (1 g each), collected in different seasons (January, March, May, July, September and November), were also extracted separately as per the procedure optimized, followed by acid hydrolysis described above. The final volume for each sample was made up to 25 ml with methanol.

Two microlitres each of the stock solution and the four working standard solutions of apigenin were applied, in triplicate, on 20×20 cm TLC plates. The developed plates were scanned at 336 nm using HPTLC densitometer. Fig. 1 shows the HPTL chromatogram of apigenin from *T. aphrodisiaca*. A standard graph was plotted against mean area under the peak and apigenin amount (μg). Aliquots (2 μl) of test samples were applied, in triplicate, to the TLC plates. Fig. 2 shows a representative HPTL chromatogram of acid hydrolyzed methanol extract of aerial parts of *T. aphrodisiaca*. The area under the curve of every sample was recorded, and apigenin content was determined from the regression equation of the standard graph.

**Fig. 1:**
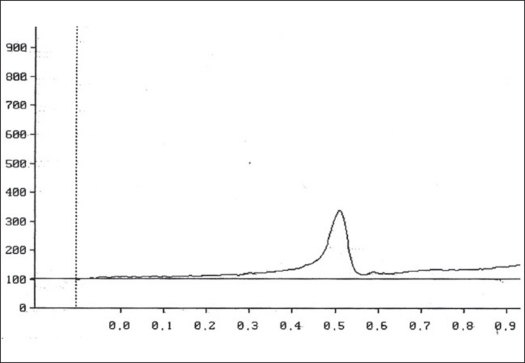
HPTL chromatogram of apigenin. HPTL chromatogram of apigenin (0.5 mg/10 ml) from *T. aphrodisiaca*. Mobile phase, toluene:ethyl acetate (4:1); visualization at 336 nm.

**Fig. 2:**
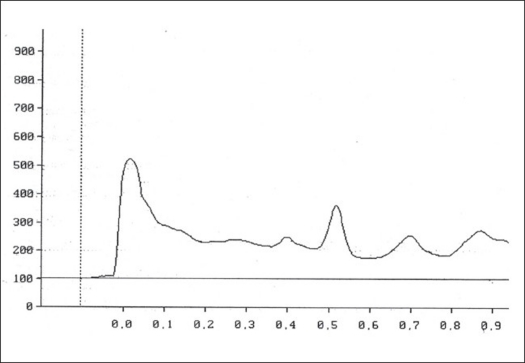
HPTL chromatogram of methanol extract of aerial parts of *T. aphrodisiaca*. HPTL chromatogram of acid hydrolyzed methanol extract of aerial parts of *T. aphrodisiaca*. Mobile phase, toluene:ethyl acetate (4:1); visualization at 336 nm.

Apigenin (0.5, 1.0 and 2.0 mg) were added to 1 g of *T. aphrodisiaca* aerial parts in three separate flasks. The extraction was carried out as per the procedure mentioned earlier. The resulting solutions were concentrated under reduced pressure, finally making up their volume to 50 ml. A 2 μl sample of every solution was subjected to HPTLC for the estimation of apigenin. The estimated quantity of apigenin was expressed as % w/w. The recovery graph was obtained by plotting % w/w-added versus % w/w-determined (fig. 3). Recovery % age was calculated by multiplying slope of the regression line by 100.

**Fig. 3:**
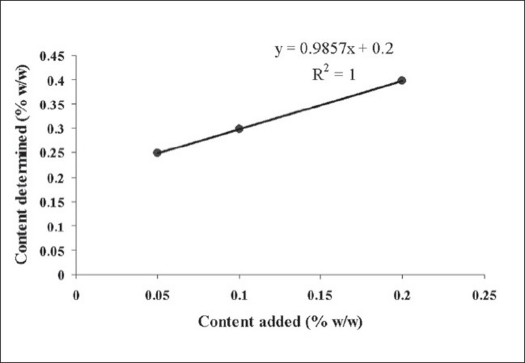
Recovery plot for apigenin. Plot of Apigenin (0.05, 0.10, 0.20 %w/w) added to *T. aphrodisiaca* aerial parts (1 g) and content of apigenin observed. Linear regression equation was y = 0.9857× + 0.2 (r^2^ =1).

Table 1 shows the content of apigenin present in aerial and segregated parts of *T. aphrodisiaca*. The content of apigenin in the marketed formulations of the plant is shown in Table 2. The content of apigenin in *T. aphrodisiaca* aerial parts, collected in different seasons, is shown in Table 3. Table 4 shows the recovery of apigenin by optimized extraction procedure.

**TABLE 1:** AMOUNT OF APIGENIN EXTRACTED FROM *T. APHRODISIACA* AERIAL AND ITS SEGREGATED PARTS

Plant part		Apigenin content (% w/w) (Mean±SD)
Direct method	Aerial parts	0.0140.001
Acid hydrolysis method	Aerial parts	0.201±0.002
	Leaves	0.214±0.002
	Stems	0.027±0.001
	Flowers	0.240±0.002
	Fruits	0.018±0.001

Each value is the mean±standard deviation of 3 determinations.

**TABLE 2:** APIGENIN CONTENT IN DIFFERENT MARKETED FORMULATIONS OF *T. APHRODISIACA* AS DETERMINED BY HPTLC DENSITOMETRY

Marketed formulation	Apigenin content (% w/v) (Mean±SD)
NLK	0.307±0.028
SBL	9.142±0.232
DWSG	11.554±0.113

Each value is the mean±standard deviation of 3 determinations.

**TABLE 3:** APIGENIN CONTENT IN *T. APHRODISIACA* AERIAL PARTS, COLLECTED IN DIFFERENT SEASONS, AS DETERMINED BY HPTLC DENSITOMETRY

Season	Apigenin content (% w/w) (Mean±SD)
January	0.141±0.001
March	0.151±0.002
May	0.176±0.002
July	0.181±0.002
September	0.199±0.002
November	0.189±0.002

Each value is the mean±standard deviation of 3 determinations.

**TABLE 4:** THE RECOVERY OF APIGENIN BY OPTIMIZED EXTRACTION PROCEDURE – VALIDATION OF HPTLC ASSAY

Apigenin			

Added content		Observed content (% w/w)	Recovery*
		
mg	% w/w	Mean^n^ ± S.D.	(% w/w)
0.5	0.05	0.249 ± 0.001	
1.0	0.10	0.299 ± 0.002	98.57
2.0	0.20	0.397 ± 0.005	

Each value is the mean±standard deviation of 3 determinations.

* slope×100

*T. aphrodisiaca* has long history of use in CNS disorders. Authors have isolated anxiolytic flavone apigenin from the plant using bioactivity-guided fractionation. In this work, apigenin content was determined in *T. aphrodisiaca* aerial and its segregated parts by HPTLC densitometry. Linearity of the calibration curve was achieved between 0.05 and 0.25 μg for apigenin (r^2^ = 0.998). Apigenin content in the hydrolyzed methanol extract of *T. aphrodisiaca* aerial parts was found to be about 14 times more than direct methanol extract (Table 1). From this observation, it can be concluded that most of the apigenin is present in *O*-glycosidic form in *T. aphrodisiaca* aerial parts. Amongst different plant parts, flowers possessed maximum content of apigenin followed by leaves. DWSG possessed maximum content followed by SBL and NLK (Table 2). Content of apigenin in DWSG was found to be about 35 times more than NLK. Despite the presence of very less content of anxiolytic component of *T. aphrodisiaca*, i.e., apigenin, NLK showed maximum anxiolytic activity at lower dose^11^. This could be probably due to: (i) addition of anxiolytic compound(s) as no official control is applicable to these OTC products, and/or (ii) improper selection of the plant material.

The effect of season on content of apigenin in *T. aphrodisiaca* was also observed (Table 3). Further, the HPTLC assay used for determining apigenin content in *T. aphrodisiaca* was validated by adding known quantities of reference apigenin to the test samples, and determining its content (fig. 3;Table 4). Recovery percentage of apigenin was observed to be very high (98.57%) indicating that the assay procedure developed, in the present investigation, is quite reliable.
